# Current views on endocytosis in filamentous fungi

**DOI:** 10.1080/21501203.2020.1741471

**Published:** 2020-03-24

**Authors:** Blake Commer, Brian D. Shaw

**Affiliations:** Department of Plant Pathology and Microbiology, Texas A&M University, College Station, TX, USA

**Keywords:** Endocytosis, sub-apical collar, filamentous fungi, FM4-64, hyphal growth, endocytic recycling

## Abstract

Filamentous fungi grow by adding cell wall and membrane exclusively at the apex of tubular structures called hyphae. Growth was previously believed to occur only through exocytosis at the Spitzenkörper, an organised body of secretory macro- and microvesicles found only in growing hyphae. More recent work has indicated that an area deemed the sub-apical collar is enriched for endocytosis and is also required for hyphal growth. It is now generally believed that polarity of filamentous fungi is achieved through the balancing of the processes of endocytosis and exocytosis at these two areas. This review is an update on the current progress and understanding surrounding the occurrence of endocytosis and its spatial regulation as they pertain to growth and pathogenicity in filamentous fungi.

## 1. Overview

Filamentous fungi (FF) uniquely produce tubular cells called hyphae in a polarised manner. This occurs through the synthesis and addition of new cell wall and membrane exclusively at the apex (Steinberg et al. 2017; Riquelme et al. 2018). The paradigm of hyphal growth has previously been to focus on exocytosis through the Spitzenkörper (SPK), an organised body of secretory macro- and microvesicles which is found only in growing fungal hyphae (Bartnicki-Garcia et al. 1989, 1995; Reynaga-Peña et al. 1997; Virag and Harris 2006). An early hypothesis proposed that the establishment of polarity is a stochastic process that autonomously transpires without the need for membrane markers (Harris 2006). However, the evidence provided since then indicates that the series of events is much less random. More recent work has forced reconsideration due to the discovery of an area that is enriched for endocytosis. This work has indicated that the area distal to the SPK, deemed the sub-apical collar, is also required for hyphal growth (Taheri-Talesh et al. 2008; Upadhyay and Shaw 2008). It is now generally understood that polarity of FF is achieved through the balancing of the processes of endocytosis and exocytosis.

**Figure 1.**
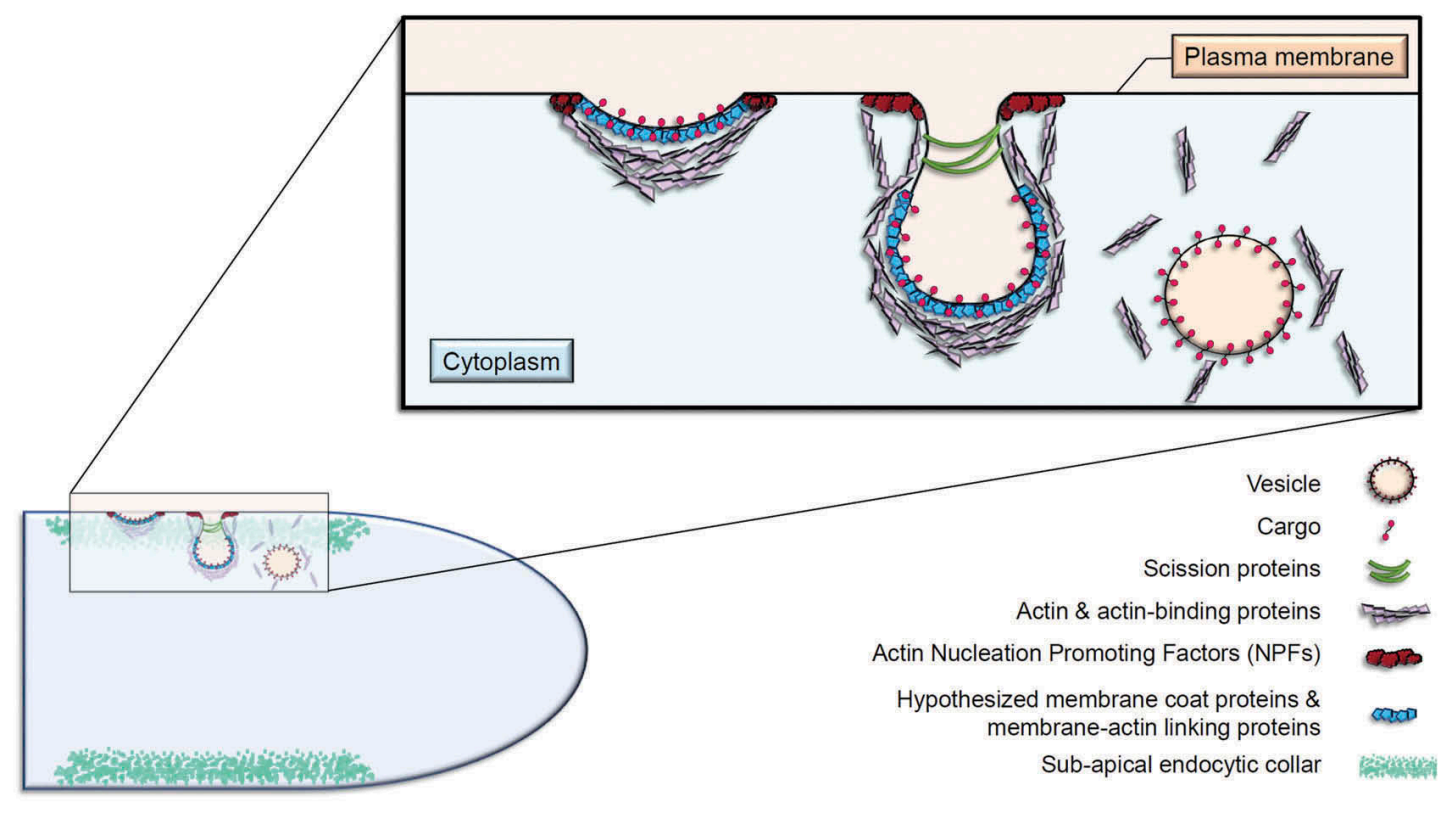
The endocytic process at the sub-apical collar of FF

Endocytosis in yeast has been thoroughly characterised (Goode et al. 2015); however, comparatively little is known about endocytosis in FF (Figure 1). In particular, little attention has been paid to proteins whose localisations and functions border between endocytosis and exocytosis in the growing hyphal apex. While a variety of process are conserved from yeasts to FF, not all components are identical, and some must be compensated for in order to afford the multiple axes of polarity and increased extension rates observed in FF. The most well-studied single-cell yeasts, *Saccharomyces cerevisiae* and *Schizosaccharomyces pombe*, also display polarised growth. However, this budding or fission growth is limited by the cell cycle, while hyphae of FF are able to continuously extend in an unrestricted unidirectional manner (Takeshita 2016). Evidence that endocytosis itself varies between yeasts and FF has also been provided through studies on clathrin localisation. While clathrin is observed both internally as well as at the cortex of *S. cerevisiae* cells, a corresponding localisation to the endocytic collar is not observed in *A. nidulans* (Newpher et al. 2005; Schultzhaus et al. 2017).

Schematic depiction of the process of endocytosis as elucidated from *Saccharomyces cerevisiae* (Lu et al. 2016). A hyphal apex is shown in the lower left corner, with a close-up partial representation of membrane bending, invagination, and scission of a vesicle. General components are highlighted as the specific proteins involved may vary from one species to another. One major difference from *S. cerevisiae* is that clathrin has been shown not to localise to endocytic sites in the sub-apical collar in some filamentous fungi (Schultzhaus et al. 2017). *Note: image was inspired by (*Lu et al. 2016*), and is not drawn to scale.*

Several reviews have summarised the various aspects of polarised growth and hyphal morphogenesis in FF over the past decade (Peñalva 2010; Harris 2011; Shaw et al. 2011; Steinberg 2014; Takeshita 2016; Steinberg et al. 2017; Riquelme et al. 2018; Verdín et al. 2019). For a more comprehensive review on the process of endocytosis itself, see (Steinberg 2014; Steinberg et al. 2017). The priority of this review is to update the current progress and understanding surrounding the occurrence of endocytosis as it pertains to growth in FF. Evidence to support the necessity of endocytosis for the maintenance of normal hyphal extension and shape in FF is provided here, as well as various theories behind its spatial regulation. Our understanding of this process as well as hyphal growth is crucial, as each one is required for the disease progression of plants, animals, and humans (Köhler et al. 2014; Zeilinger et al. 2016; Riquelme et al. 2018).

## 2. Does endocytosis occur in filamentous fungi?

**Figure 2.**
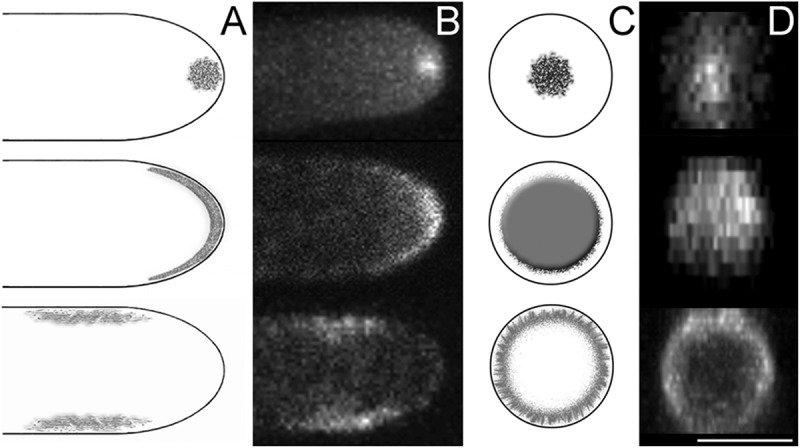
Three apical areas to which proteins associated with membrane turnover localise

It has been demonstrated that many proteins associated with membrane turnover (such as adapter proteins, cargoes, endocytic machinery, etc.) localise to three different apical regions of growing hyphae: the SPK, the apical crescent, and the sub-apical collar (Figure 2) (Upadhyay and Shaw 2008; Araujo-Bazan et al. 2008; Sudbery 2011). Since endocytosis is a vital life process in FF, it is imperative that it is evaluated based on studies which implement live cell imaging. Fluorescently tagging proteins to evaluate localisation patterns was a rather arduous process at the turn of the century, and it still can be in many organisms, particularly those without sequenced or publicly available genomes. Alternatively, select vital stains or dyes that had been created for use in live cell imaging were instrumental in allowing more comprehensive studies at the time. It was through the use of these dyes that endocytosis was first discovered in FF (Hoffmann and Mendgen 1998). The former belief behind the mechanisms of hyphal growth was based solely on the notion of exocytosis, which predominantly occurs through the SPK. However, FM4-64 staining, combined with the discovery of the sub-apical collar and genomic data, forced re-evaluation of the commonly accepted notion of the vesicle supply centre.

(a) Schematic depiction of the three areas associated with endocytosis at the apices of growing hyphae, viewed from the side. (A, top) The Spitzenkörper (SPK) stains readily with the vital stain FM4-64, suggesting that it functions in the recycling of endocytic vesicles that are produced at the hyphal tip. (A, middle) Cell-end markers line the apical crescent, or dome, and localise growth machinery to hyphal tips. (A, bottom) The sub-apical collar is an area enriched for endocytosis where apical recycling takes place. (b) Fluorescence images of each of the three areas, represented by *Aspergillus nidulans* DnfB::GFP (Schultzhaus et al. 2015), HapA::GFP (unpublished data), and EcoA::GFP (unpublished data), respectively. (c-d) Schematic depiction & fluorescence images of the three areas as viewed from the apex of the hypha. *Scale bar 2.5 µm.*

### 2.1. History of markers for endocytosis

Several membrane-selective dyes and markers have previously been utilised in order to investigate the countless processes and pathways occurring within living fungi. For example, Lucifer Yellow, TMA-DPH, FITC-dextran, FM1-43, and FM4-64 were all utilised in an attempt to visualise internalisation and subsequent trafficking (Fischer-Parton et al. 2000; Peñalva 2005). Of the various dyes used, the lipophilic marker dye FM4-64 was particularly instrumental in revolutionising the current understanding of endocytosis in FF.

FM4-64 was initially shown to be endocytosed at the plasma membrane in *S. cerevisiae* (Vida and Emr 1995), and it subsequently became a vital stain which was, and still is, considered a marker for endocytosis in the yeasts. This discovery was soon-after followed by the application of FM4-64 to FF. In *Uromyces fabae*, FM4-64 uptake was observed within seconds of its application, and the most intense localisation was to the membrane at the hyphal apex, making it the first evidence for endocytosis in a fungal germ tube (Hoffmann and Mendgen 1998). This sparked an interest in using it to study FF, and shortly thereafter, endocytosis was also demonstrated in ungerminated and germinated spores of the FF *M. grisea* (Atkinson et al. 2002). In addition to labelling the apical hyphal membrane, FM4-64 uptake was also observed in the SPK (Fischer-Parton et al. 2000). This evidence was rather controversial, particularly because the SPK was believed to be involved only with apical secretion through Golgi-derived exocytic vesicles (Reynaga-Peña et al. 1997). These new data, however, indicated that the SPK functions additionally in recycling endocytic vesicles produced at the hyphal tip (Upadhyay and Shaw 2008). Furthermore, it suggested that endocytosis is also vital for hyphal growth, which has since been corroborated by numerous studies.

In the years following this discovery, FM4-64 was applied to a multitude of filamentous species (Fischer-Parton et al. 2000; Atkinson et al. 2002). While the use of FM4-64 in FF was still relatively limited in the early 2000s (Peñalva 2005), its use is now considered standard practice for experiments involving endocytic or apically localised proteins in FF.

### 2.2. The discovery of the sub-apical collar

The evidence provided from various experiments involving FM4-64 made it clear that the internalisation of membranes also contributes to apical growth. This was subsequently reinforced by the discovery of the sub-apical collar in *A. nidulans* (Taheri-Talesh et al. 2008; Upadhyay and Shaw 2008; Araujo-Bazan et al. 2008), which is an area enriched for endocytosis that lies 1–5 µm distal to the SPK. The location of the collar in the sub-apex is maintained over time during hyphal growth, and dissipates with the cessation of growth, similar to the maintenance of the SPK. Prior to this discovery, it was primarily believed that fungal growth occurred due to secretion through the SPK.

### 2.3. Theories regarding the need for endocytosis in the collar

It was previously shown that endocytosis in *S. pombe* is spatially associated with the actin cytoskeleton during polarised growth (Gachet and Hyams 2005). Therefore, it does not come as a surprise that the “zone of endocytosis” in FF also happens to be where actin is predominantly and intensely localised (Upadhyay and Shaw 2008; Delgado-Alvarez et al. 2010; Schultzhaus et al. 2016). However, it is still unclear why endocytosis is enriched in the sub-apical collar. At this time, several theories have been proposed to elucidate the reasoning behind the presence and spatial regulation of the collar in FF. The original hypothesis for tip growth proposed that the SPK was comprised of Golgi-derived exocytic vesicles which were destined to fuse with the apex. However, this vesicle supply centre concept needed to be expanded to account for the function of endocytosis at the apex and sub-apical collar once it was identified in FF (Gierz and Bartnicki-Garcia 2001).

One explanation is provided by the apical recycling model, which indicates endocytosis at the sub-apical collar maintains the polarisation of apically localised membrane proteins (Shaw et al. 2011; Hernandez-Gonzalez et al. 2018). These membrane proteins would in turn be marking areas of polarisation at the apices of hyphae, and they would be displaced along the membrane during growth. These proteins would therefore need to then be removed by the sub-apical collar in order for the hyphoid shape to be maintained.

Recently, another hypothesis suggested that endocytosis in the collar acts as a means for removing excess secreted plasma membrane, which has recently been quantified in *Neurospora crassa* (Riquelme et al. 2018; Bartnicki-Garcia et al. 2018). Based on the data provided by Bartnicki-Garcia et al. in 2018, an estimated 9800 vesicles per minute are needed in order to maintain hyphal growth and cell wall expansion. This is likely on the higher end compared to many other FF due to the large size and fast growth rate of *N. crassa* hyphae. Nonetheless, the number of vesicles discharged is estimated, on the lower end, to be around 59,000 vesicles per minute (Bartnicki-Garcia et al. 2018). Some of these proteins must therefore be recycled at the sub-apical collar in turn. In this model, endocytosis acts primarily to remove the excess membrane provided through secretion, and does so efficiently due to its close proximity to the apex. While the idea of removing excess plasma membrane was proposed as an explanation for reducing the sheer quantity of secreted membrane, it also parallels the apical recycling model.

Another comparable hypothesis is that the apex is lined with proteins which localise to the plasma membrane and are deemed “cell-end markers” or “polarity markers.” Cell-end markers, such as *A. nidulans* KipA, TeaA, and TeaR, have been shown to be involved in localising growth machinery to hyphal tips (Konzack et al. 2005; Takeshita et al. 2013). These polarised proteins are cytoskeleton-dependent, and subsequently direct cell polarity. Secretion at the hyphal apex dilutes the accumulation of polarity markers, creating a cyclical process of assembling and disseminating cell-end markers in a pulse-like pattern (Takeshita 2016). This proposed circulation of proteins also bares striking similarities to the apical recycling model. These are not mutually exclusive theories, as each one was proposed through the perspective of a different area at the hyphal apex.

## 3. Experimental evidence

Regardless of the reasoning for the location and maintenance of the sub-apical collar in FF, there is now little doubt about its existence. Countless experiments have shown that a sub-apical area enriched for endocytosis arises during growth. Further studies have provided evidence for the recycling of various polarised proteins. For example, the essential *A. nidulans* chitin-synthase ChsB is polarised through indirect endocytic recycling, which involves both exocytosis at the apex and endocytosis at the sub-apical collar. ChsB is subsequently trafficked to the TGN cisternae and then ultimately re-delivered to the apex (Hernandez-Gonzalez et al. 2018). Additional evidence is discussed in more detail below.

### 3.1. *Fimbrin mutant in* Aspergillus

The processes of endocytosis and exocytosis are synchronised and regularly maintain a spatial and temporal relationship during growth. Evidence for this relationship has been provided in the form of multiple mutational studies. When a gene involved in one of the two processes is disrupted, so too is normal hyphal elongation and the uniquely notable hyphoid shape. For example, when the *A. nidulans* F-actin cross-linking gene and endocytic marker fimbrin (*fimA*) was disrupted, cells displayed abnormal isotropic swelling and an inability to otherwise maintain polarity. Furthermore, FimA and the secretory vesicle marker GsaA were fused with mCherry and localised using the conditional *niiA* promoter in *A. nidulans*. Aberrant growth was evident when endocytosis was disrupted, and neither the SPK nor the standard zone of endocytosis at the collar could be seen (Upadhyay and Shaw 2008). This evidence highlighted the partnership of these two associated processes, and the aforementioned apical recycling model was proposed as a result of these findings.

### 3.2. NPFxD motif

One of only two endocytic signal sequences discovered in yeast to date is the NPFX_(1,2)_D motif (and the similar but less effective variations, DPFxD, NPF, or DPF), which was shown to mark proteins for endocytosis and is required for polarity in yeast (Tan et al. 1996; Howard et al. 2002; Piao et al. 2007). This motif is found in multiple membrane proteins, and is recognised by the adapter protein Sla1p through an interaction with the SHD1 (Sla1p Homology Domain) region (Costa et al. 2005). Mutating one or more of the initial three residues in this motif to Alanine (A) has been shown to halt endocytic uptake of the protein (Tan et al. 1996). Studies have shown that the contribution of the motif to the function and localisation of specific endocytic cargo proteins (Liu et al. 2007; Schultzhaus et al. 2015). For example, yeast proteins Drs2p and Dnf1p each contain at least one NPFxD motif. In wild-type (WT) cells, Drs2p localises to the *trans*-Golgi network (TGN) and Dnf1p localises both to the plasma membrane and to endo-membranes. When the NPFxD-dependent endocytic mechanisms were inactivated, the localisation of each protein was altered (Liu et al. 2007). To further test the motif, as well as the apical recycling model, the orthologous NPFxD-containing protein DnfA in *A. nidulans* was also mutated. DnfA::GFP displayed polarised localisation to the apical plasma membrane and the SPK; however, this polarised localisation was also lost upon mutating the motif to AAFxD (Schultzhaus et al. 2015). The re-localisation of DnfA::GFP upon disruption of the motif corroborates the notion that apical recycling maintains the polarisation of the protein. These studies also reinforce the proposition that the peptide motif is essential for proper endocytic uptake in a variety of cargo proteins.

### 3.3. The requirement of endocytosis for pathogenicity

Recent studies have also provided evidence that the endocytic pathway is important for pathogenicity in many plant pathogens. For example, a multitude of proteins in *Magnaporthe oryzae* have been shown to be involved in both endocytosis and pathogenicity. It was recently found that *M. oryzae* protein MoEnd3 is critical for development and virulence due to its role in mediating receptor endocytosis (Li et al. 2017b). Additionally, MoRab5A and MoRab5B, both of which are Rab5 homologs in *M. oryzae*, are critical for endocytosis and plant pathogenesis, among other things (Yang et al. 2017). Other studies have linked MoArk1, MoAbp1, MoAct1, and MoCAP proteins (among others) to endocytosis, growth, the actin cytoskeleton, and therefore, pathogenicity (Li et al. 2017a, 2019). Investigation of *Ustilago maydis* protein Yup1 similarly demonstrated that endocytosis is essential for the beginning stages of pathogenesis (Fuchs et al. 2006). This requirement has been further demonstrated in multiple species of *Fusarium* as well. For example, *Fusarium graminearum* proteins FgMon1 and FgRab7 have been shown to play critical roles in modulating vesicle trafficking, endocytosis, and plant infection (Li et al. 2015). Deletions of FgSnc1, FgSnx41, or FgSnx4 also affect endosomal sorting, polarised growth, and ultimately pathogenicity (Zheng et al. 2018).

### 3.4. Genome-wide studies & bioinformatic analysis

An invaluable tool made widely available to fungal biologists in recent years is bioinformatic analysis. Combined with genome-wide studies, these analyses allow for evolutionary relationships and dynamics to be revealed and reconsidered. Examples of this include the recently elucidated origin of fungal hyphae and adaptation mechanisms in dimorphic fungi (Muñoz et al. 2018; Kiss et al. 2019). Trends within and between organisms have been made visible with the availability of databases of information, and the field has been able to utilise this information to expand the current understanding of signalling networks and orthologous relationships. Bioinformatic analysis of fungal genomes has also provided overwhelming evidence that endocytosis and endocytic recycling are important life processes for FF (Baker 2006; Jorgensen et al. 2009, 2010; De Souza et al. 2013; Ramsubramaniam et al. 2014). For example, sequencing and annotating the *A. nidulans* genome revealed that there were many more genes implicated in vegetative growth and morphogenesis than in yeast (Harris et al. 2009). In addition, genomic studies using expressed sequence tag (EST) analyses and whole-genome sequencing in *A. oryzae* have elucidated genes involved in growth and secretion (Abe et al. 2006). In *A. niger*, a transcriptomic fingerprint was also developed for apical branching and hyphal elongation (Meyer et al. 2009).

## 4. Conclusions & outstanding questions

Both endocytosis and the spatial coupling of endocytosis with secretion have now been shown to be of great importance for hyphal extension in several FF. In the last two decades, our understanding of how FF grow has been entirely revolutionised by the discovery of endocytosis and the areas in which it occurs. Since then, many questions have been answered, and even more have been posed.

Single particle tracking, fluorescence recovery after photobleaching (FRAP), and various other advanced microscopy techniques have provided a means for scientists to answer pressing questions about endocytosis. For example, data on the quantification of endocytosis was previously very limited (Thilo 1985); however, Bartnicki *et. al* were able to implement a comprehensive FRAP protocol in 2018 and better estimate the number of vesicles that are endocytosed in the sub-apical collars of *N. crassa* hyphae. This impressive milestone serves as a baseline on which others in the field can build. This also answered multiple questions posed by Peñalva *et. al* in 2010 regarding whether or not the apex contains an excess of membrane for which endocytosis compensates. It was found that, while endocytosis does partially compensate for the excess membrane, there is still far more membrane produced at the apex than endocytosis can account for. This result was somewhat unexpected and suggests that perhaps these secreted proteins function in additional ways that we have yet to identify. It is possible that there are additional routes of endocytosis that are not adequately measured by this method, or that the overwhelming amounts of molecules which are recycled cannot be captured in their entirety by FRAP data alone.

The understanding of the secretory network at the hyphal tip has progressed significantly in recent years. However, comparably little has focused on the process of internalisation, which has been shown to be equally necessary for hyphal growth and polarity, and is the hallmark characteristic of FF. Now that it is generally accepted that endocytosis does occur in FF, priority should be given to identifying the proteins that act as cargo for endocytosis, or that participate in apical recycling. A reverse genetics approach to identify genes which encode the endocytosis peptide motif NPFxD is currently underway. In addition, whole genome sequencing provides an opportunity to identify genes from mutant collections created in the classical genetics era that are involved in endocytosis.

It was also recently suggested that “endocytic components are underexplored targets for engineering fungal cell factories” (Cairns et al. 2019). Many species of fungi are industrially utilised and harvested in mass for useful molecules such as enzymes, proteins, secondary metabolites, and organic acids (Cairns et al. 2018, 2019). Since many enzymes and molecules are secreted at the apex, the amount produced tends to initially correspond to the number of hyphal tips. However, an increased number of hyphal tips does not always correlate to an elevation in protein concentration. A recent explanation for this is that unconventional protein secretion pathways are activated during the fermentation process, meaning secretion is no longer predominantly occurring at the apex, but instead through the cell membrane or septa (Veiter et al. 2018). Little work has been done to investigate the role of endocytosis in the fermentation process, even though we know that the processes of exocytosis and endocytosis are tightly coupled. It is entirely possible that the decrease in overall secretion could instead be due to an increase in endocytosis, or an alteration of the specific cargo which is endocytosed. The preference for recycled products could fluctuate with various environmental conditions, such as diauxic shifts, for example. If this is elucidated, the fermentation environment could potentially be manipulated or optimised based on an ideal organismal ratio, or by first increasing endocytosis.

Furthermore, the dynamic localisation of GFP-fusion proteins must continue to be tested. Researchers should take advantage of organisms that cooperate in large-scale studies so that curated databases such as FungiDB can consolidate and distribute these data (Stajich et al. 2012). Not only do we need to characterise and localise multiple proteins, but we also need to investigate the relationships among them and focus on those whose roles vary between yeast-like and hyphal growth. Since many pathogenic fungi are dimorphic, priority should also be given to signalling networks and proteins that do not follow the typical yeast model of life and growth, or that are specific to FF altogether.

As the availability and affordability of advanced technology increases, so too will the discoveries in this field. The development of advanced microscopic tools and methods for bioinformatic analysis will continue to significantly contribute to our current knowledge on filamentous fungal growth, as well as its interconnectedness with the environment.
